# Novel HDAC inhibitor Chidamide synergizes with Rituximab to inhibit diffuse large B-cell lymphoma tumour growth by upregulating CD20

**DOI:** 10.1038/s41419-019-2210-0

**Published:** 2020-01-06

**Authors:** Xu-Wen Guan, Hua-Qing Wang, Wei-Wei Ban, Zhi Chang, Hai-Zhu Chen, Li Jia, Feng-Ting Liu

**Affiliations:** 1 Department of Hematology and Oncology, Tianjin Union Medical Center, 300191 Tianjin, China; 2 Tianjin Medical University, Tianjin, China; 3 Tianjin Medical University Cancer Institute and Hospital, National Clinical Research Center for Cancer, Key Laboratory of Cancer Prevention and Therapy, Tianjin, China; 4 Tianjin’s Clinical Research Center for Cancer, Tianjin, China; 5 Centre for Haemato-Oncology, Barts Cancer Institute, Queen Mary University of London, London, UK

**Keywords:** B-cell lymphoma, Preclinical research

## Abstract

Loss of CD20 is a major obstacle for the retreatment of relapsed/refractory diffuse large B cell lymphoma (DLBCL) with Rituximab-associated regimens. Histone deacetylation causes gene silencing and inhibits CD20 expression. Chidamide is a novel inhibitor for histone deacetylases (HDACs). We hypothesize that Chidamide could overcome Rituximab-mediated down-regulation of CD20 and facilitate Rituximab-induced killing. In this study, we determine the mechanism of synergy of Chidamide with Rituximab in DLBCL using in vitro and in vivo models. We found that the levels of CD20 protein surface expression on five DLBCL cell lines were significantly and positively correlated with the sensitivities of cells to Rituximab. Treatment with Rituximab significantly reduced CD20 surface expression at the protein levels. RNA sequencing showed that Chidamide significantly increased expression of more than 2000 transcriptomes in DLBCL cells, around 1000 transcriptomes belong to the cell membrane and cell periphery pathways, including MS4A1. Chidamide significantly increased CD20 surface expression in DLBCL cell lines. Combination with Chidamide significantly synergized Rituximab-induced cell death in vitro and significantly inhibited tumour growth in DLBCL-bearing xenograft mice. A patient with relapsed/refractory DLBCL achieved a complete response after three cycles combined treatment with Chidamide and Rituximab. In conclusion, our data demonstrate for the first time that inhibition of HDACs by Chidamide significantly enhanced Rituximab-induced tumour growth inhibition in vitro and in vivo. We propose that CD20 surface expression should be used clinically to evaluate treatment response in patients with DLBCL. Chidamide is a promising sensitizer for the retreatment of DLBCL with Rituximab.

## Introduction

Diffuse large B-cell lymphoma (DLBCL) is the most aggressive type of non-Hodgins lymphoma worldwide. Treatment with anthracycline-based chemotherapy regimens such as a combination of cyclophosphamide, doxorubicin, vincristine and prednisone (CHOP) plus Rituximab immunotherapy (R-CHOP) has improved overall survival (OS) in patients with DLBCL by 10–15%, compared to treated with CHOP alone^1^. However, about 30–50% DLBCL patients are not cured by this treatment regimen^2^. Relapsed/refractory DLBCL after R-CHOP is difficult to salvage and the challenge is to develop effective and personalized strategies^3^. The mechanism by which DLBCL patients develop resistance to R-CHOP is currently unclear and understanding the molecular basis of this treatment failure is crucial for improving clinical outcome of DLBCL patients.

Rituximab is a chimeric monoclonal antibody targeted against the pan-B-cell marker CD20. Binding of Rituximab to CD20 is not sufficient to kill all lymphoma cells, indicating there are mechanisms of resistance^4^. The loss of CD20 expression was observed following Rituximab treatment in a subset of patients, which may cause treatment failure for Rituximab retreatment^5–8^. There were cases of CD20-deficient lymphoma relapses identified following treatment with Rituximab-associated regimens in DLBCL^6^. Rituximab-induced downregulation of CD20 expression is mainly due to deacetylation of histones by histone deacetylases (HDACs)^9–11^, internalization of CD20 molecule^12^ and loss of CD20/Rituximab complex from cell surface^13^. Insufficient surface CD20 protein affects Rituximab-induced lipid raft domain organization and downstream signalling, leading to Rituximab resistance^14^. Studies have shown that acetylated histones promoted the binding of transcription factors to DNA by reducing the affinity of DNA and loosening the chromatin structure^15^. H3K27ac is a histone modification associated with active enhancers^16,17^. The enhancer regions of MS4A1 (CD20) in DLBCL cells are H3K27ac^18^. Upregulation of CD20 expression by either specific inhibitors for HDAC6 (Tubacin and Ricolinostat) or non-specific HDAC inhibitors (Valproic acid and Romidepsin) showed sensitizing potential in Rituximab-induced cell death in malignant B cells^9–11^.

HDACs play important roles in cancer development by regulating the expression and activity of numerous proteins involved in cancer initiation and progression^19^. Currently, only four HDAC inhibitors, Vorinostat, Romidepsin, Panobinostat and Belinostat are licensed in oncology for the treatment of cutaneous T cell lymphoma^20–22^. A phase II clinical trial study showed that combination Rituximab with Vorinostat exhibits inhibitory effect on disease progression in indolent B cell non-Hodgkin lymphoma with an acceptable safety profile and durable responses to HDAC inhibitor^23^.

Chidamide is a novel and orally active benzamide class of HDAC inhibitor that selectively inhibits activity of HDAC1, 2, 3 and 10 (refs. ^24–26^). It has been approved by China Food and Drug Administration in 2015 for the treatment of relapsed/refractory peripheral T cell lymphoma^27,28^. One case report showed that combination of Chidamide with R-CHOP exhibited complete response (CR) in a relapsed/refractory DLBCL patient^29^. We hypothesize that Chidamide may facilitate the therapeutic efficacy of Rituximab in DLBCL by upregulation of CD20 expression.

In this study, we aimed to determine the potency and the molecular mechanism of action of Chidamide on DLBCL cells and whether Chidamide synergizes Rituximab-induced tumour growth inhibition. Chidamide or Rituximab-mediated changes in transcriptomes of DLBCL cells were conducted using RNA-seq. The roles of Chidamide or Rituximab on CD20 expression and tumour growth inhibition were determined in vitro and in vivo.

## Results

### Rituximab downregulates CD20 protein expression in human DLBCL cells

**Fig. 1:**
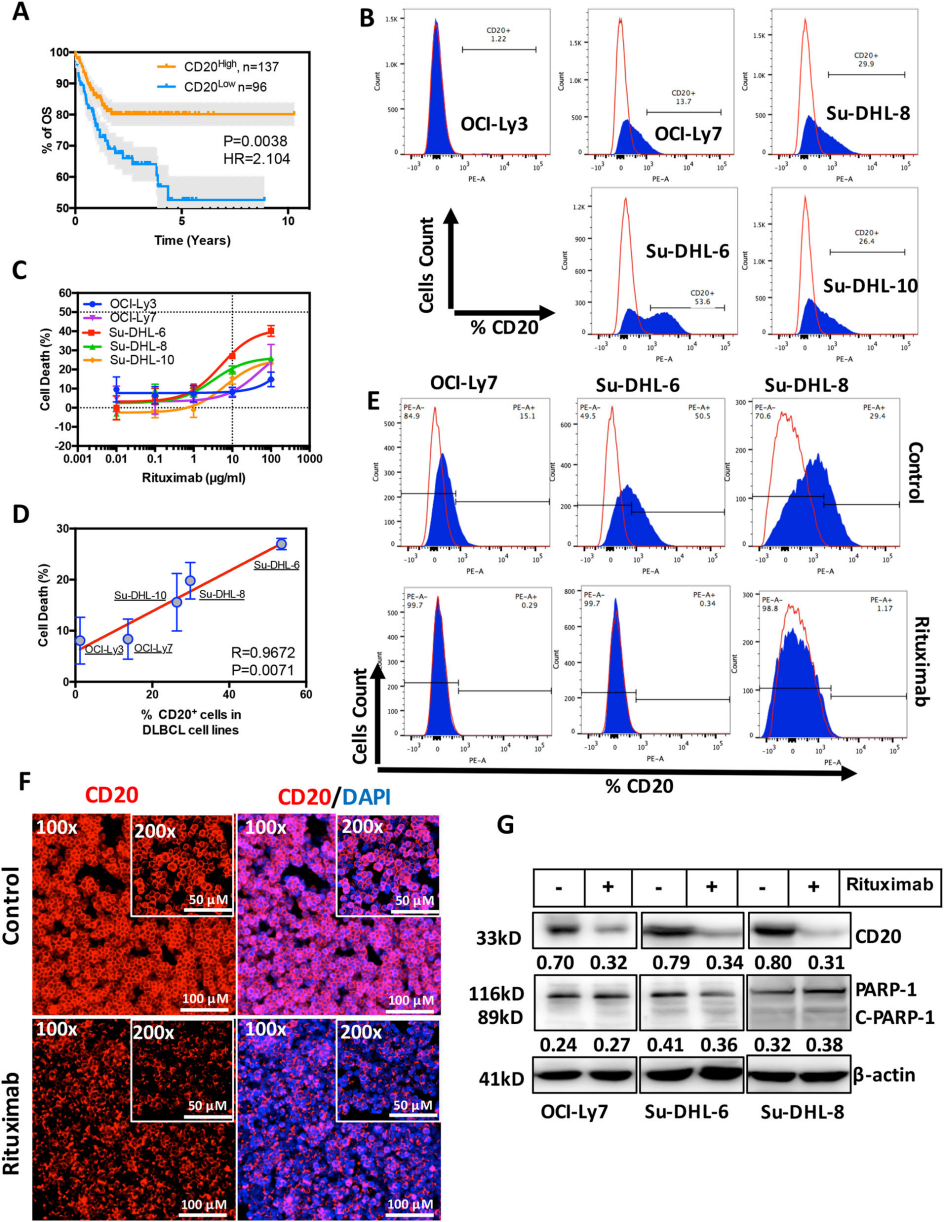
Effect of Rituximab in CD20 expression in human DLBCL cells. **a** Kaplan Meier overall survival (OS) curves of patients with DLBCL. Two subgroups, CD20^High^ and CD20^Low^ mRNA expression from DLBCL1 (R-CHOP treated, *n* = 233) were defined according to the cut-off point generated by X-tile software. **b** Determination of CD20 surface expression on five DLBCL cell lines (OCI-Ly3 OCI-Ly7, Su-DHL6, Su-DHL8 and Su-DHL10) by flow cytometry. Cells were stained with anti-CD20-PE antibody (blue peaks) or mouse IgG-PE control isotype (red lines). **c** Five DLBCL cell lines were treated with 0.01, 0.1, 1, 10 and 100 µg/ml of Rituximab or PBS for 24 h and percentages of cell death were determined by MTT test. Data shown are mean ± SD from three independent experiments. **d** Correlation between surface CD20 expression and sensitivity of DLBCL to Rituximab-induced cytotoxicity. Correlation were analysed by the Pearson product–moment correlation. *R* = correlation coefficient. Data shown are mean ± SD from three independent experiments. **e** Determination of CD20 surface expression by flow cytometry. DLBCL cell lines, OCI-Ly7, Su-DHL6 and Su-DHL8, were treated with Rituximab (10 µg/ml) for 24 h. Cells were stained with anti-CD20-PE antibody (blue peaks) or mouse IgG-PE control isotype (red lines). **f** Immunofluorescent staining of CD20. After treatment with Rituximab, cells on slides were fixed and stained with CD20-PE antibody (Red). DAPI (Blue) indicates nuclear localization. Original magnification ×100 and ×200. **g** Detection of protein expression by western blotting. After treatment with Rituximab (10 µg/ml) for 24 h, CD20 expression and PARP-1 cleavage was determined by western blotting. Fifty micrograms of proteins were loaded onto each lane. FL-PARP-1 and C-PARP-1 indicate full length and cleaved PARP-1, respectively. β-Actin was used as a loading control. Numbers under each lane were ratios of specific protein to β-actin. Data presented were from at least three independent experiments.

Treatment with R-CHOP has significantly improved the life expectancy in DLBCL patients compared with using CHOP alone (Supplementary Fig. 1a). Levels of CD20 (MS4A1) mRNA expression was retrospectively analysed in 233 DLBCL patients who were previously treated with R-CHOP. Lower expression of CD20 significantly confers inferior clinical outcome in DLBCL patients after treatment with R-CHOP (Fig. 1a). CD20 expression has no effect on the clinical outcome in DLBCL patients treated with CHOP (data not shown). The levels of CD20 surface expression differ among five DLBCL cell lines, as detected by flow cytometry (Fig. 1b). Accordingly, the cell lines with lower CD20 expression are resistant to Rituximab (Fig. 1c). The sensitivity of DLBCL cells to Rituximab is significantly and positively correlated to the levels of CD20 expression (*R* = 0.9672, *P* = 0.0071; Fig. 1d). Treatment with 10 µg/ml Rituximab for 24 h downregulated CD20 expression in DLBCL cells (Fig. 1e–g) but there is no remarkable sign of apoptotic cell death (cleavage of PARP) in this settings (Fig. 1g). CD20 mRNA levels were not altered by Rituximab, determined by RT-PCR (Supplementary Fig. 1b), indicating that Rituximab-mediated downregulation of CD20 is not at the transcriptional level. Expression of other B-cell marker, such as CD19, was not affected by Rituximab (Supplementary Fig. 1c), suggesting that Rituximab-induced downregulation of surface markers may be specific to CD20. These results demonstrate that the levels of CD20 are crucial for the treatment response of DLBCL patients to Rituximab. Rituximab per se downregulates CD20 protein expression which could lead to retreatment failure with Rituximab.

### Chidamide is a potent inhibitor for HDACs in DLBCL cells

**Fig. 2:**
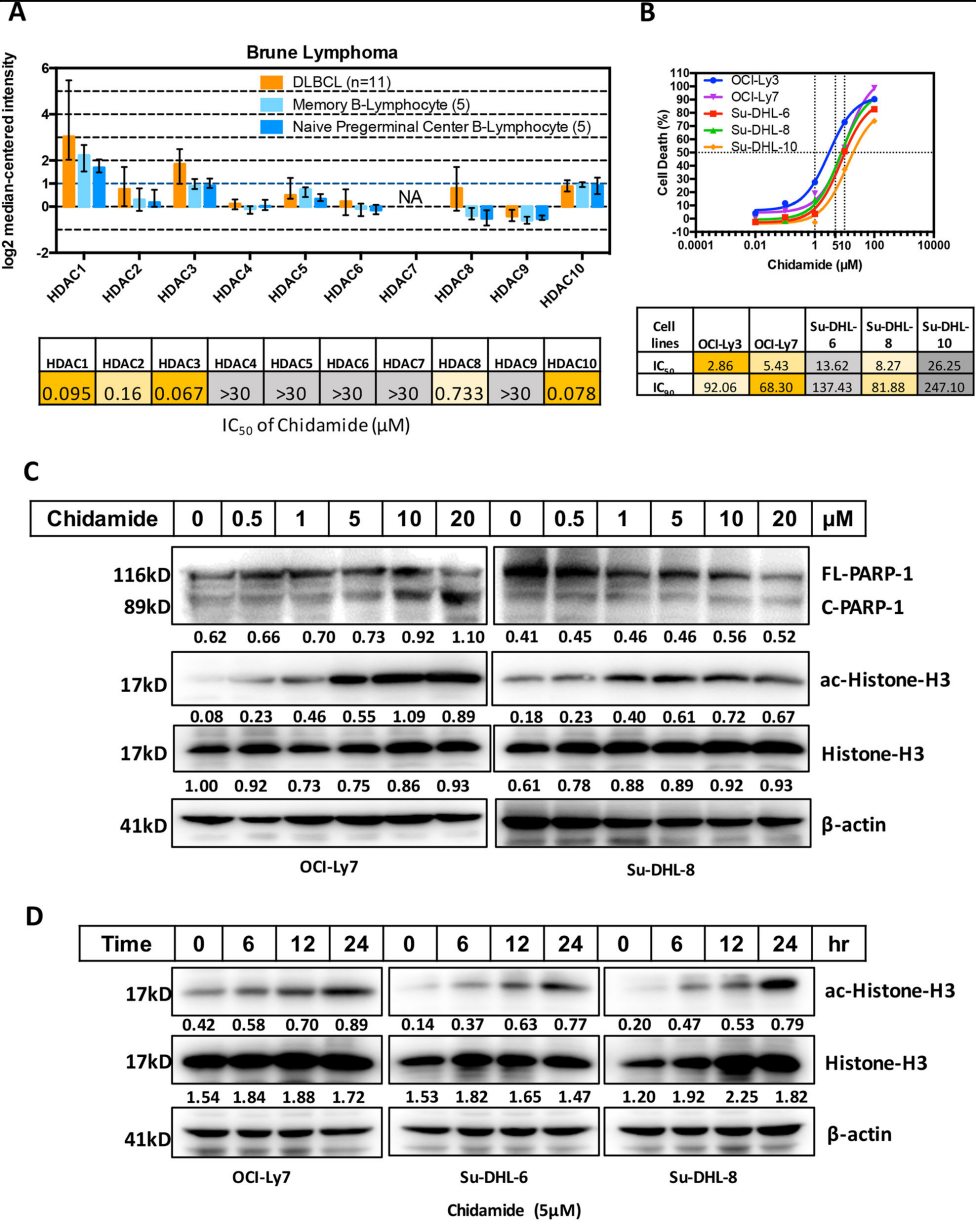
Effects of Chidamide on DLBCL cells. **a** Comparison differences of HDAC1-10 mRNA expression between DLBCL tissues and normal B cells (top). Data were obtained from GEO database (GSE12453). IC_50_s of Chidamide against recombinant human HDACs (bottom). **b** Five DLBCL cell lines were treated with 0.01, 0.1, 1, 10, 100 µM of Chidamide or DMSO for 24 h and percentages of cell death were determined by the MTT test. (top) Data shown are mean ± SD from three independent experiments. IC_50_s and IC_90_s of DLBCL cell lines to the treatment of Chidamide were calculated with the linear regression based on MTT data (bottom). **c** Effect of different dose of Chidamide on PARP-1 cleavage, Histone-H3 acetylation and expression of Histone-H3. DLBCL cell lines, OCI-Ly7 and Su-DHL8, were treated with indicated dose of Chidamide for 24 h, the levels of FL-PARP-1 and C-PARP-1, ac-Histone-H3 (Lys27) and Histone-H3 expression were determined by western blotting. Numbers under each lane were ratios of specific protein to β-actin. **d** Time-course of Chidamide on Histone-H3 acetylation and expression of Histone-H3. DLBCL cell lines, OCI-Ly7, Su-DHL6 and Su-DHL8 were treated with 5 µM Chidamide for indicated hours, the levels of ac-Histone-H3 (Lys27) and Histone-H3 expression were determined by western blotting. Numbers under each lane were ratios of specific protein to β-actin. Data presented were from at least three independent experiments.

There are increased expression of HDAC 1, 2, 3 and 10 mRNA in DLBCL tissues compared with normal B lymphocytes^30^. However, the pathological roles of such an increased in HDAC expression in DLBCL have not been clinically investigated. Chidamide is a selective HDAC inhibitor for these sub-types^24^ (Fig. 2a). The cytotoxicity of Chidamide was determined in five DLBCL cell lines by MTT test. The IC_50_ data showed that DLBCL cell lines OCI-Ly3 and OCI-Ly7 which have lower expression of CD20 are more sensitive to Chidamide-induced cell death (Fig. 2b). Five doses of Chidamide were tested on the level of histone acetylation by western blotting and the results showed that 5 µM of Chidamide is the most effective dose to induce histone acetylation but had no effect on apoptotic cell death (Fig. 2c). Increase in Chidamide concentration (higher than 5 µM) did not further increase the level of histone-H3 acetylation. Chidamide-mediated histone acetylation is time-dependent and 24 h treatment showed the most effective prominent (Fig. 2d). These results suggest that Chidamide is potent histone deacetylase inhibitor in DLBCL.

### Chidamide upregulates transcriptomes in hematopoietic lineage, including CD20 mRNA

**Fig. 3:**
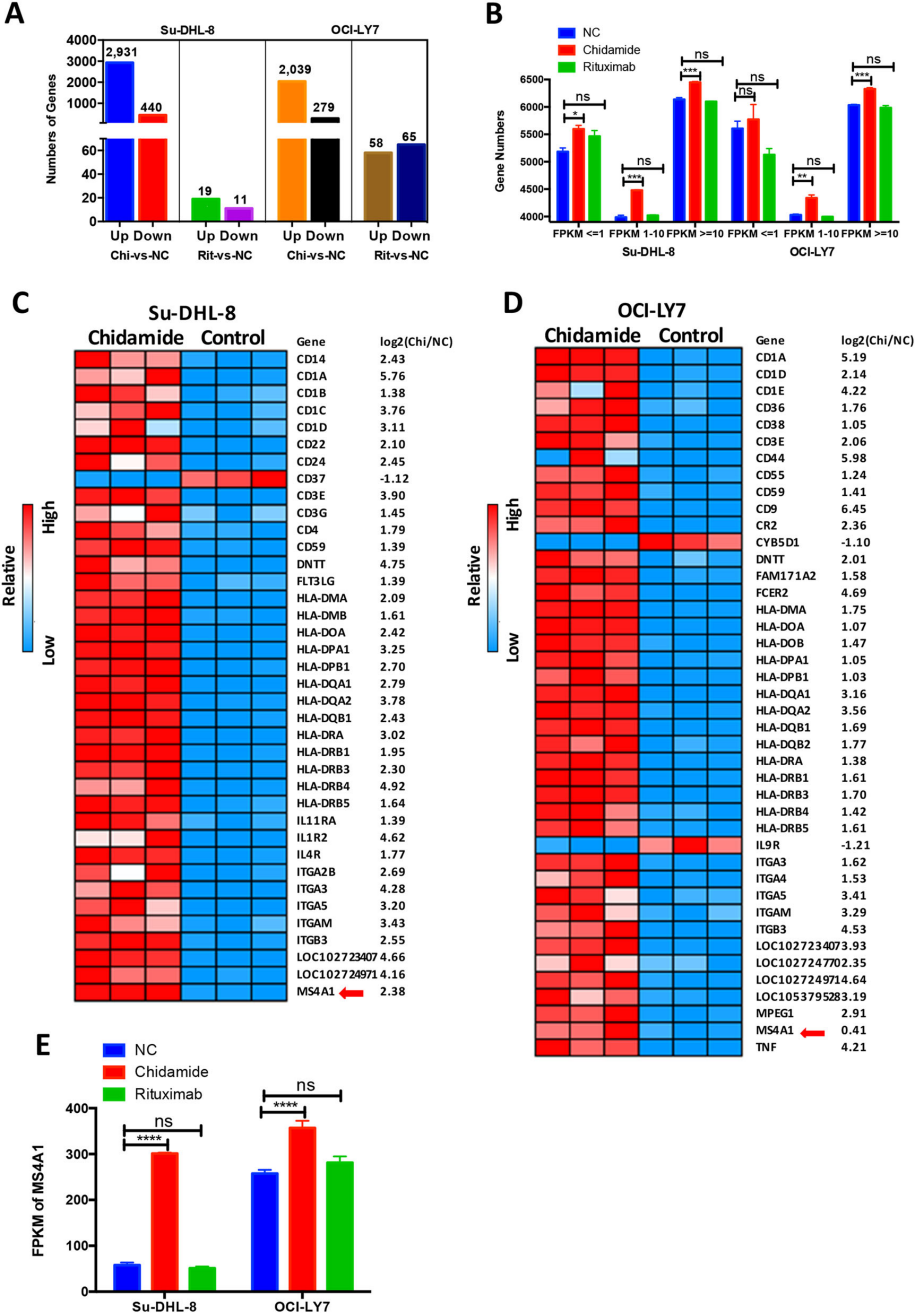
Effects of Chidamide and Rituximab in cellular transcriptomes of DLBCL cells. **a** Numbers of genes upregulated (Up) or downregulated (Down) more than twofold than untreated control (NC) after treatment with Chidamide (Chi) or Rituximab (Rit) for 24 h. **b** Number of genes in different FPKM (fragments per kilobase million) interval (FPKM ≤ 1, 1–10 FPKM, FPKM ≥ 10) in different groups (NC, Chidamide and Rituximab). **c**, **d** Heatmap of the differential expression genes (DEGs) in hematopoietic cell lineage pathway (Kyoto Encyclopaedia of Genes and Genomes pathway (KEGG)) in response to Chidamide for 24 h. **c** Su-DHL8 cell line and **d** OCI-Ly7 cell line. The red arrow points CD20 mRNA, MS4-A1. **e** Effects of Chidamide or Rituximab in MS4A1 (CD20 mRNA) expression. The levels of MS4A1 expression was represented as numbers of FPKFs. ‘n.s.’ indicates not significant.

Histone deacetylation induced by HDACs leads to gene silencing. We determined whether Chidamide could modulate gene expression, such as CD20, by performing unbiased transcriptome profiling using RNA seq. Su-DHL-8 and OCI-Ly7 cell lines were treated with either 5 µM Chidamide or 10 µg/ml Rituximab for 24 h. Totally, expression of 3371 genes in Su-DHL8 and 2381 genes in OCI-Ly7 was modulated by Chidamide. Among them, 2931 genes in Su-DHL8 and 2039 genes in OCI-Ly7 were upregulated. However, only 30 genes in Su-DHL8 and 123 genes in OCI-Ly7 were modulated by Rituximab (Fig. 3a). Chidamide but not Rituximab significantly increased expression on genes with lower FPKM (≈1–10) and middle-high FPKM numbers (FPKM>10) in both cell lines. Significantly increased expression in genes with very low FPMK numbers (≤1) was only observed in the Su-DHL8 cell line (Fig. 3b). In the hematopoietic lineage pathway, 37 differentially expressed genes (DEGs) in Su-DHL8 and 39 DEGs in OCI-Ly7 were significantly up-regulated by Chidamide, mainly cluster of differentiation (CD) family, including CD20 (MS4A1), human leucocyte antigens (HLA) and integrin (ITGs). More than 10 HLA genes were upregulated, indicating the positive effect of Chidamide on antigen presentation. Chidamide induced upregulation of CD20 (MS4A1) gene in both Su-DHL8 and OCI-Ly7 cells. Expression of ITGA-3 (CD49c), ITGA-5 (CD49e), ITGB-3 (CD61), ITGMG (CD11b), CD1A, CD3E, CD1D, CD59, LOC102723407 (immunoglobulin heavy variable 4-38-2-like protein) and LOC102724971 (putative V-set and immunoglobulin domain-containing-like protein) genes were significantly increased by Chidamide in both cell lines (Fig. 3c, d). Chidamide significantly increased CD20 expression in both cell lines but Rituximab did not show effect on CD20 mRNA expression (Fig. 3e), in a good agreement with our RT-PCR results.

### Chidamide upregulates transcriptomes in multiple signalling pathways in DLBCL cells

**Fig. 4:**
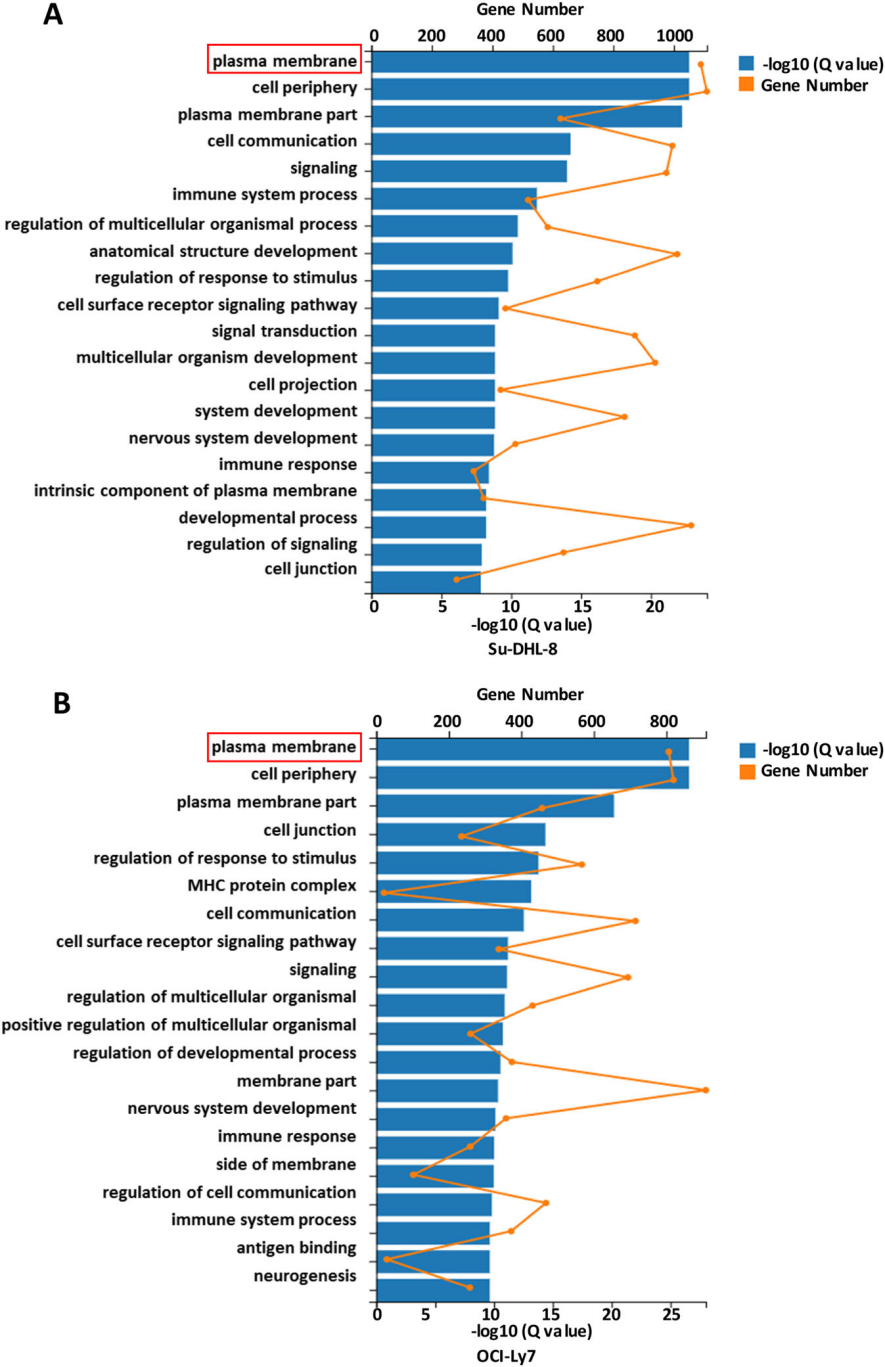
Gene Ontology (GO) enrichment analysis of Chidamide-induced modification in DEGs. Gene Ontology (GO) enrichment analysis of DEGs was analysed by Gene Set Enrichment Analysis (GSEA). **a** Pathways affected by Chidamide in the Su-DHL8 cell line. **b** Pathways affected by Chidamide in the OCI-Ly7 cell line.

Using Gene Set Enrichment Analysis (GSEA) of DEGs in gene ontology (GO), Chidamide-modulated genes in both DLBCL cell lines were categorized into multiple pathways. Among these categories, 1109 genes in Su-DHL8 cells and 909 genes in OCI-Ly7 cells responded to Chidamide belong to the cell membrane and cell periphery pathways. Nine hundred and two genes in Su-DHL8 and 687 genes in OCI-Ly7 cells which belong to cell communication, regulation of response to stimuli and regulation of multicellular organism development categories were regulated by Chidamide (Fig. 4a, b). These results demonstrate that HDACs control expression of multiple transcriptomes in DLBCL cells. Inhibition of HDACs by Chidamide effectively modulated expression of mRNAs which encode cell membrane proteins.

### Expression of HDAC2 and HDAC3 are associated with inferior clinical outcome in patients with DLBCL

To determine which HDCA isotype is overexpressed in DLBCL and associated with inferior clinical outcome in patients with DLBCL, HDAC 1–10 mRNA expression was retrospectively analysed in DLBCL1 (R-CHOP treated) and DLBCL2 (CHOP-treated) cohorts. HDAC2 is consistently overexpressed in both DLBCL1 and DLBCL2 cohorts and higher expression of HDAC2 is significantly associated with inferior clinical outcome in DLBCL patients (*P* = 0.0125 and HR = 1.915 in DLBCL1 cohort; *P* = 0.0002 and HR = 2.318 in DLBCL2 cohort), regardless of treatment regimens. HDAC3 is also consistently overexpressed in both cohorts but is not significantly associated with OS, although HRs are higher than 1 in both cohorts (HR = 1.324 and 1.325, respectively). In contrast, lower expression of HDAC6 is significantly and consistently associated with poor clinical outcome in both DLBCL1 and DLBCL2 cohorts (Supplementary Fig. 2a, b). The expressions of all HDACs is significantly correlated with MS4A1, with HDAC1, 2, 8 and 9 are positively correlated and HDAC3–7 and HDAC10 are negatively correlated with MS4A1 (Supplementary Table 3). Downregulation of CD20 could be associated with higher expression of HDACs which is negatively correlated with MS4A1 gene expression. The nuclear localization of HDAC is essential for regulating gene transcription. Only HDAC1–3 and 8 are exclusively expressed in the nucleus^31^, HDAC6 and 10 are expressed in the cytoplasm, and HDAC4, 5, 7 and 9 are expressed in both cytoplasm and nucleus^32^ (Supplementary Table 3). These data demonstrate that higher expression of HDAC2 and 3 is associated with poor clinical outcome in DLBCL patients. Increased expression of nuclear HDAC3 is negatively correlated with reduced expression of MS4A1, suggesting that downregulation of CD20 may be due, at least partly, to increased expression of HDAC3 in DLBCL. Targeting HDAC2 and HDAC3 by HDAC inhibitor may improve clinical outcomes in DLBCL patients.

### Chidamide overcomes Rituximab-induced CD20 downregulation

**Fig. 5:**
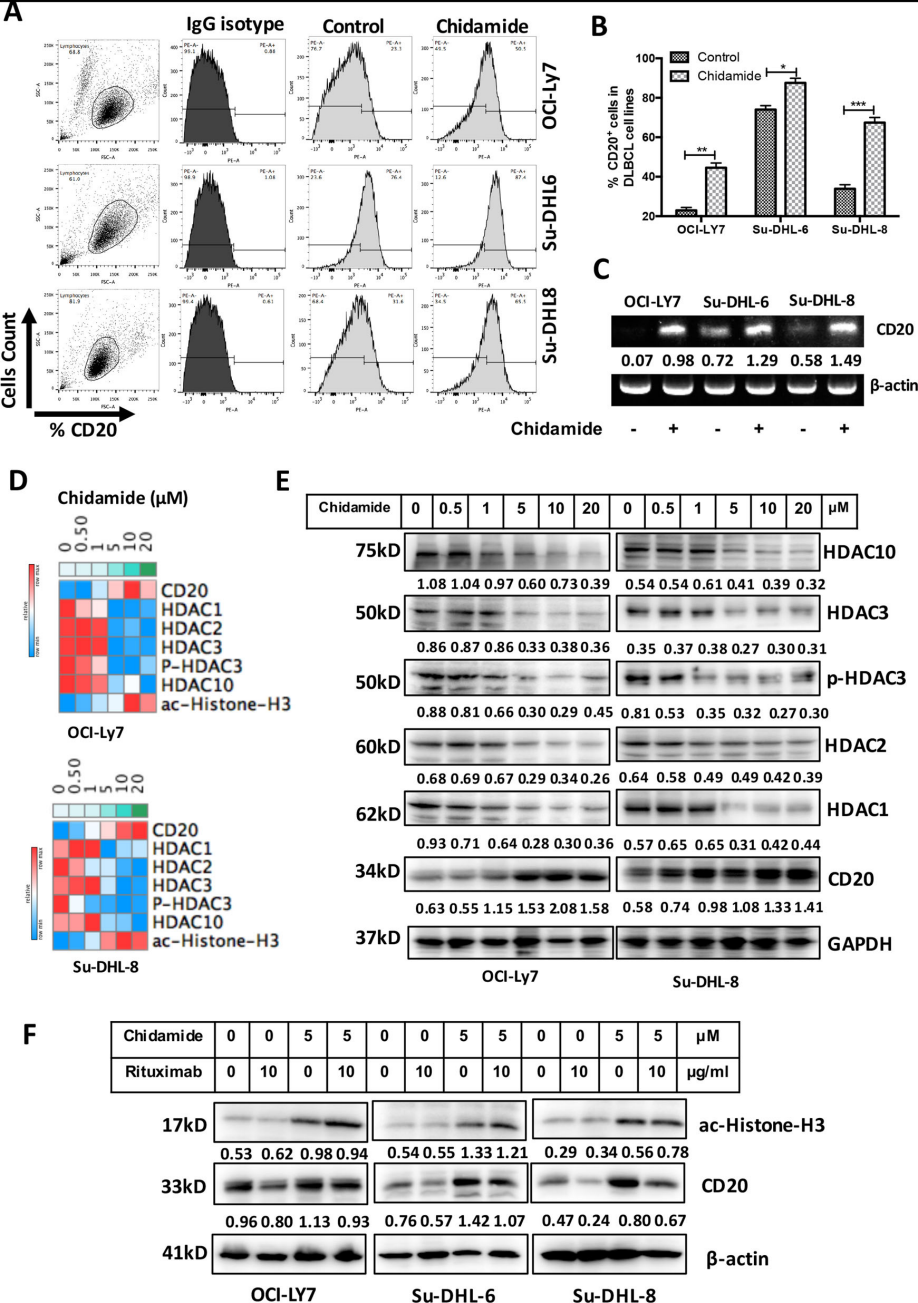
Effect of Chidamide on CD20 expression in DLCBL cells. **a**, **b** CD20 surface expression. DLBCL cell lines were treated with 5 µM Chidamide for 24 h and CD20 surface expression was determined by flow cytometry. Data shown are mean ± SD from three independent experiments. **c** CD20 mRNA expression. DLBCL cells were treated with 5 µM Chidamide for 24 h. Levels of CD20 mRNA expression were determined by RT-PCR. Numbers under each lane were ratios of CD20 mRNA to β-actin mRNA. **d** Effect of Chidamide on protein expression. DLBCL cells were treated with indicated dose of Chidamide for 24 h. The levels of HDAC1, HDAC2, HDAC3, p-HDAC3 (Ser424), HDAC10 and CD20 expression were determined by western blotting. Numbers under each lane were ratios of specific protein to GAPDH. **e** Effects of Chidamide and/or Rituximab on CD20 and ac-Histone-H3 (Lys27) protein expression. After treatment with 5 µM Chidamide and/or 10 µg/ml Rituximab, CD20 and ac-Histone-H3 (Lys27) expression was determined by western blotting. β-Actin was used as a loading control. Numbers under each lane were ratios of specific protein to β-actin. Data presented were from at least three independent experiments.

We examined whether treatment with Chidamide increases CD20 expression. DLBCL cell lines were treated with 5 µM Chidamide for 24 h. Chidamide significantly increased CD20 surface expression in DLBCL cells (Fig. 5a, b). Chidamide-mediated increase in CD20 mRNA expression was confirmed by RT-PCR (Fig. 5c). Chidamide-induced downregulation of HDAC1, 2, 3 and 10 and phosphorylated HDAC3 (p-HDAC3), upregulation of CD20, and acetylated histone-H3 were in dose-dependent manners (Fig. 5d), indicating that deacetylation process is inhibited by Chidamide. Combined treatment with Chidamide and Rituximab overcomes Rituximab-induced downregulation of CD20 (Fig. 5f). These results demonstrate that treatment with Chidamide inhibits expression of HDAC1, 2, 3, 10 and p-HDAC3. Upregulated expression of CD20 by Chidamide is due to restoration of the acetylation of histone-H3 which was inhibited by HDACs.

### Chidamide synergizes Rituximab-mediated cytotoxicity

**Fig. 6:**
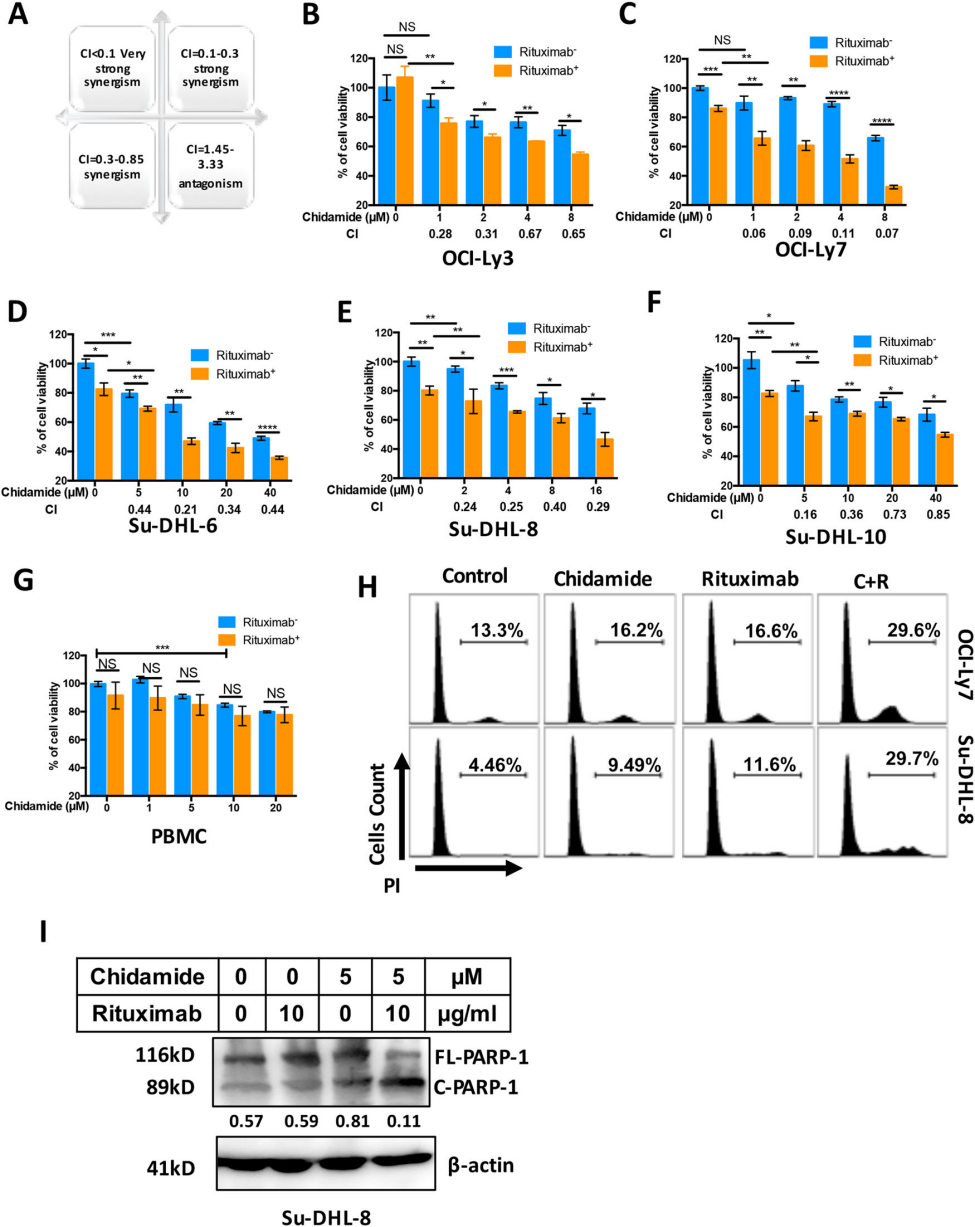
Synergism between Chidamide and Rituximab in DLBCL cells. **a** Schema of combination index (CI). **b**–**f** Synergistic effect of Chidamide on Rituximab-induced cytotoxicity. Cytotoxicity was determined in five DLBCL cell lines. Cells were treated with indicated dose of Chidamide combined with or without 10 µg/ml of Rituximab for 24 h and percentages of cell death (cytotoxicity) were determined by MTT test. Data shown are mean ± SD from three independent experiments. Numbers under each plot were combination index (CI). **g** Cytotoxic effect of Chidamide and/or Rituximab on normal PBMC. PBMCs were isolated from five normal donors and treated with Chidamide and/or Rituximab for 24 h. **h** Synergistic effect of Chidamide on Rituximab-induced cell death. Cells were treated with 5 µM Chidamide combined with or without 10 µg/ml of Rituximab for 24 h. Cell death was measured by flow cytometry after staining PI. The percentages of dead cells in different cell cycle were shown in the plots. **i** Synergistic effect of Chidamide and Rituximab on PARP-1 cleavage. Cells were treated with 5 µM Chidamide combined with or without 10 µg/ml of Rituximab for 24 h. the levels of FL-PARP-1 and C-PARP-1 were determined by western blotting. β-Actin was used as a loading control. Numbers under each lane were ratios of specific protein to β-actin. Data presented were from at least three independent experiments.

We next determined whether Chidamide could synergize Rituximab-mediated cytotoxicity in five DLBCL cell lines using MTT test. The levels of synergism and combination index (CI) were calculated using CompuSyn software (Fig. 6a). The IC_50_s of Chidamide to each cell line are OCI-Ly3 = 2.86 µM; OCI-Ly7 = 5.43 µM; Su-DHL-6 = 13.62 µM; Su-DHL-8 = 8.27 µM; and Su-DHL-10 = 26.25 µM (Fig. 2a). Among five DLBCL cell lines, the OCI-Ly3 and OCI-Ly7 cell line are more sensitive and the Su-DHL6 and Su-DHL-10 cell line are relatively resistant to Chidamide. Four different doses of Chidamide were designed according to the sensitivities of cell lines to Chidamide. DLBCL cell lines were treated with 10 µg/ml Rituximab. OCI-Ly3 and OCI-Ly7 cell lines were co-treated with 1, 2, 4 and 8 µM Chidamide. A strong synergy (CI = 0.28) and a very strong synergy (CI < 0.1) were shown in OCI-Ly3 and OCI-Ly7 cell lines treated with 1 µM Chidamide and Rituximab (Fig. 6b, c). The Su-DHL-8 cell line was co-treated with 2, 4, 8 and 16 µM Chidamide and showed strong synergies with Rituximab (CI < 0.3) (Fig. 6e). Su-DHL6 and Su-DHL10 cell lines were co-treated with 5, 10, 20 and 40 µM Chidamide and 5 µM Chidamide in Su-DHL10 cells and 10 µM Chidamide in Su-DHL6 cells had strong synergies with Rituximab (Fig. 6d, f). The cytotoxic effects of Chidamide/Rituximab were tested in normal peripheral blood mononuclear cells (PBMC). After 24 h treatment, Chidamide dose higher than 10 µM showed significant cytotoxic effect on normal cells (Fig. 6g). Chidamide plus Rituximab-induced cell death in DLBCL cell lines was also confirmed using propidium iodide (PI) staining and detected by flow cytometry (Fig. 6h) and apoptotic cell death detected by PARP cleavage (Fig. 6i). These results indicate that Chidamide strongly synergizes Rituximab-mediated cytotoxicity in DLBCL cell in vitro.

### Chidamide facilitates Rituximab-induced tumour growth inhibition in DLBCL xenograft mice

**Fig. 7:**
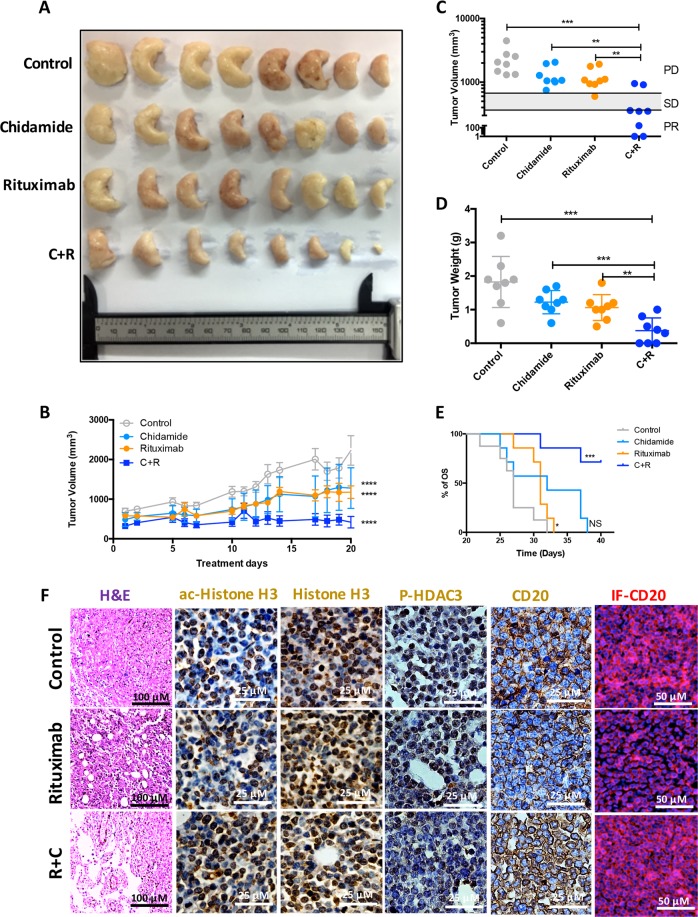
Effect of Chidamide on Rituximab-induced tumour growth inhibition xenograft mice. **a** A collection of DLBCL tumours from human DLBCL tumour-bearing xenograft mice after the endpoints of each indicated treatments (*n* = 32). **b**, **c** Comparison of tumour volumes. Human DLBCL (OCI-Ly7)-bearing BALB/c nude mice were treated with Chidamide and Rituximab according to dosing schedules. Tumour volume was measured, calculated daily and presented as mean volume ± SD. Comparison between final and initial tumour volumes was used to evaluate treatment responses. CR complete response, PR partial response, SD stable disease, and PD progressive disease. **d** Comparison of tumour weights. Data shown are mean ± SD. **e** Kaplan Meier overall survival (OS) curves of tumour-bearing xenograft mice. Four subgroups, vehicle control treatment (*n* = 8), Chidamide treatment (*n* = 8), Rituximab treatment (*n* = 8) and Chidamide + Rituximab treatment (*n* = 8) from two independent experiments. C + R means combined treatment with both Chidamide and Rituximab. **f** Counteracting effect of Chidamide on Rituximab-induced down-regulation of CD20. Tumour samples were fixed and sliced and stained with H&E (the left column); histochemical chemistry staining of ac-Histone H3, Histone H3, P-HDAC3 and CD20 (the middle columns); immunofluorescent staining of CD20 (the right column). Images were taken with a Nikon microscope (original magnification ×100, ×200 and ×400). Data presented were from three independent experiments.

To determine whether Chidamide enhances Rituximab-mediated DLBCL tumour growth inhibition in vivo, 6-week-old female BALB/c nude mice were inoculated with human DLBCL OCI-Ly7 cells. After 3 weeks, tumour-bearing xenograft mice were treated with Rituximab and/or Chidamide daily for another 3 weeks. The dose of Rituximab for in vivo use is 200 µg/mice, equivalent to 10 µg/ml. The dose of Chidamide for mice is 3.9 mg/kg, equivalent to 10 µM. Tumours were collected and fixed at the endpoints for mice (Fig. 7a). Treatment with Chidamide or Rituximab alone significantly reduced DLBCL tumour volumes but combination with both Chidamide and Rituximab showed a maximum inhibitory effect on tumour growth (Fig. 7b, d). Treatment with both Chidamide and Rituximab produced 2/8 CR and 4/8 partial response (PR) (Fig. 7c). Importantly, the combined treatment significantly prolonged survival of DLBCL-bearing mice (Fig. 7e). Treatment with Rituximab reduced CD20 expression and the levels of CD20 were restored by combination Chidamide and Rituximab (Fig. 7f). Rituximab alone has no effect on the level of histone-H3 acetylation and HDAC3 phosphorylation. When combined with Chidamide, the treatment remarkably increased the level of histone-H3 acetylation and decreased the levels of phosphor-HDAC3 in mice DLBCL tissues (Fig. 7f). These results suggest that combination Chidamide with Rituximab overcome resistance of DLBCL cells to Rituximab by upregulation of CD20 expression.

### Combined treatment with Chidamide and Rituximab for a patient with relapsed/refractory DLBCL

**Fig. 8:**
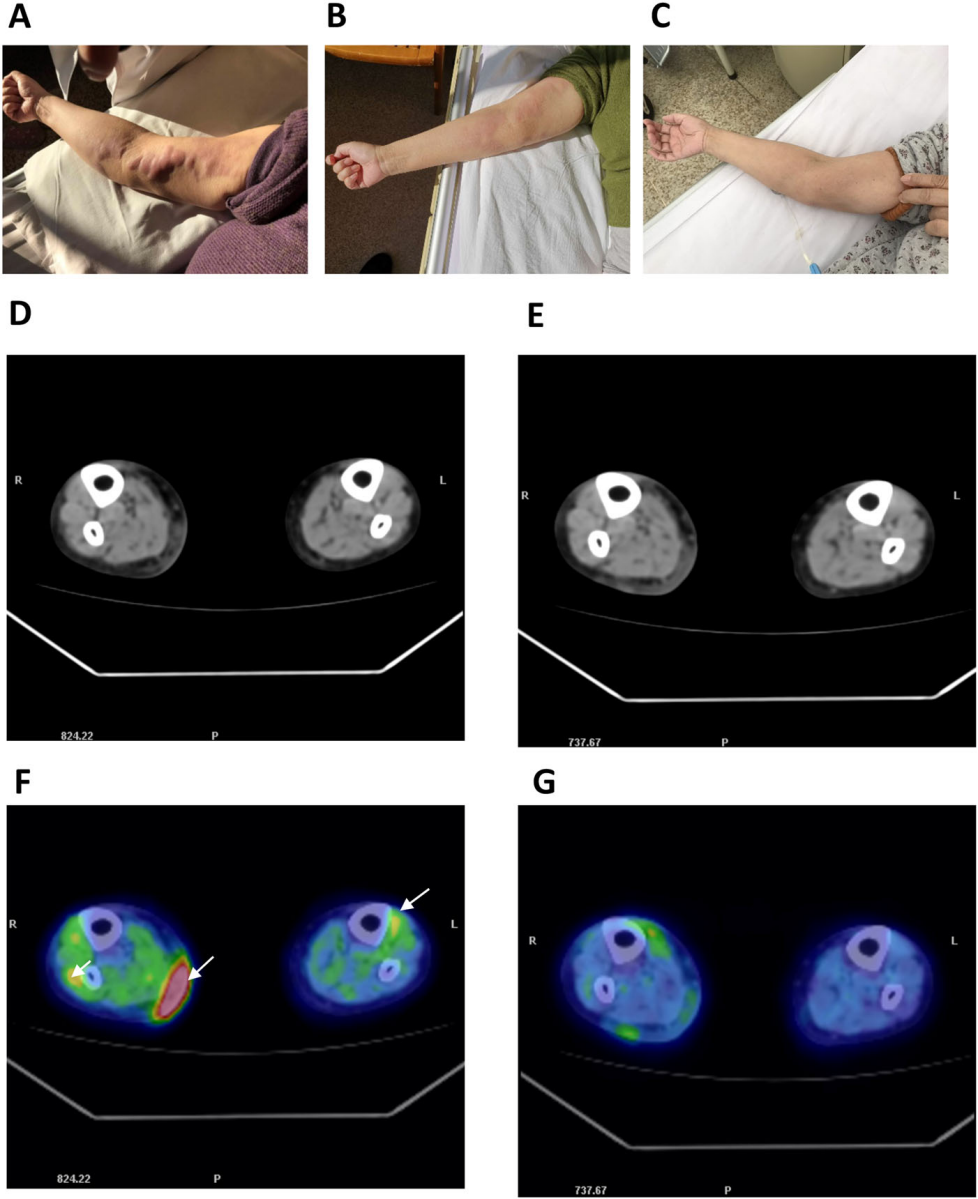
Comparison of DLBCL cutaneous lesions before and after treatment with Chidamide plus Rituximab. **a**–**c** Visual results, taken by camera. **d**, **e** CT images. **f**, **g** PET-CT images. **a**, **d**, **f** Images of patient with multiple cutaneous lesions before the treatment with Chidamide plus Rituximab. The arrows in ‘F’ indicate high-density masses under the skin and in the muscles.

In a clinical study, a 74-year-old women with relapsed/refractory DLBCL had a multiple cutaneous lesion recurrence (Fig. 8a, d, f) and showed no further response to the treatment with Rituximab-associated regimens. This patient was treated with Chidamide plus Rituximab for three cycles. After the first cycle, the visual observation showed partial respond, as the skin lesions in her right arm almost disappeared (Fig. 7b). After the third cycle, the patient achieved a CR to the treatment, as that visual observation and PET-CT images showed that the skin lesions in her arm and legs completely disappeared (Fig. 8c, e, g). This result demonstrates that combination of Chidamide with Rituximab may be a potent strategy for the treatment of relapsed/refractory DLBCL.

## Discussion

In this study, we identified a distinct mechanism of synergy between Chidamide and Rituximab in the treatment of DLBCL in vivo and in vitro leading to an effective control of DLBCL tumour growth. Treatment with Rituximab causes loss of CD20 protein which leads to retreatment failure for DLBCL patients. Chidamide, a novel HDAC inhibitor, increases expression of many membrane proteins, including CD20, and significantly synergized Rituximab-induced tumour growth inhibition in vitro and in vivo.

Aberrant down-modulation of CD20 protein expression in DLBCL after treatment with Rituximab has been observed in the clinical settings^33–35^. Our results showed that Rituximab-mediated downregulation of CD20 is at the protein levels because the levels of CD20 mRNA expression sustained after treatment with Rituximab. The precise mechanism by which Rituximab-mediated loss of CD20 protein is elusive. It was reported that Rituximab induces CD20 internalization. We observed that treatment with Rituximab leads to depletion of CD20 from the cells. This suggests that internalized CD20-containing vesicles may be digested by enzymes in the lysosomes.

Lower expression of CD20 mRNA is significantly associated with worse clinical outcome in DLBCL patients in response to R-CHOP. Decreased CD20 mRNA expression is not a consequence of the treatment with Rituximab, suggesting that it is caused by HDAC-mediated gene silencing. CD20 mRNA expression is negatively correlated with levels of HDAC3, 4, 5, 6, 7 and 10 isoforms. However, only higher levels of HDAC2, 3 and 5 are associated with poor clinical outcomes. Inhibition of HDAC2 and 3 by Chidamide could control disease progression of DLBCL. CD20 expression may be controlled by HDAC3 but not HDAC2 because levels of HDAC2 and CD20 mRNA expression are positively correlated in DLBCL patients. Chidamide effectively inhibited expression of HDAC1, HDAC2, HDAC3 and HDAC10 and phosphorylated HDAC3 protein expression which is negatively associated with accumulation of acetylated histone-H3 and CD20 expression.

The integrity of lipid raft is crucial for Rituximab/CD20-mediated cytotoxicity^36^. Plasma membrane proteins and lipids are both crucial for dynamic assembly of cell membrane rafts and cellular signal transduction^37,38^. RNA seq results showed that expression of over 2000 mRNAs was upregulated by Chidamide and about half of those upregulated mRNAs belong to plasma membrane pathways. Expression of five subunits of integrins was upregulated by Chidamide. Integrins are required for raft/cytoskeleton formation and internalization process^39,40^. Increase in membrane protein expression, such as integrins, may lead to membrane remodelling and facilitate Rituximab-mediated raft assembling and therefore enhance the efficacy of Rituximab. In addition, Chidamide mediates upregulation of tumour antigen presentation which could have potential for facilitating checkpoint inhibitor therapy^41^.

We focused the role of Chidamide on CD20 expression and related gene expression in DLBCL cell lines and found that 5 µM Chidamide could sufficiently induce the expression of the acetylated histone-H3 expression and reduce the expression of HDAC1, 2, 3, 10 and p-HDAC3 (Ser424), and increase in Chidamide concentration (higher than 5 µM) did not further increase or decrease the expression of these genes. Chidamide at 5 µM significantly increased CD20 expression at both mRNA and protein levels. We therefore co-treated DLBCL cell lines with 5 µM Chidamide and 10 µM Rituximab. Co-treatment of DLBCL cell lines with both Chidamide and Rituximab counteracted Rituximab-induced loss of CD20 protein. This phenomenon has been verified in DLBCL xenograft mice tumour tissue.

We tested the sensitivities of DLBCL cells to Chidamide-induced cytotoxicity in five DLBCL cell lines. OCI-Ly3, OCI-Ly7 and Su-DHL8 cell lines are more sensitive (IC_50_ 2.86–8.27 µM) and Su-DHL6 and Su-DHL10 cell lines are more resistant (IC50 13.62-26.25 µM) to Chidamide. However, the IC50 of Chidamide on enzyme inhibition of HDAC1, 2, 3 and 10 are 0.095, 0.160, 0.067 and 0.078 µM, respectively^24^. This indicates that there are some off-target effects of Chidamide in its cytotoxic effect. Rituximab at 10 µg/ml induced about 20% cell death in Su-DHL6, 8 and 10 but it is less effective to the OCI-Ly3 and OCI-Ly7 cell line which expresses the lowest level of CD20 protein. We used three different doses of Chidamide combining with 10 µg/ml Rituximab to test for synergy. These combinations showed either a very strong synergy in the OCI-Ly7 cell line or strong synergy in other four cell lines on the lowest doses of Chidamide for each cell line.

The IC_50_ of Chidamide on DLBCL cell lines is around 10 µM; hence, the daily dose of Chidamide for OCI-Ly7-bearing mice was used as 3.9 mg/kg, equivalent to 10 µM. The mice showed well-tolerated to this treatment without significant weight loss during the course of treatment. Treatment with Chidamide or Rituximab alone was not able to control tumour growth in DLBCL-bearing mice. However, combination of Chidamide with Rituximab effectively inhibited tumour growth and significantly prolonged survival rate of tumour-bearing mice. As expected, this combined regimen increased the level of ac-histone-H3 and decreased that of phopho-HDAC3, as a result retained the level of CD20 in the tumour tissue. This further confirmed that the synergistic effect of Chidamide on Rituximab is at least partly due to maintain sufficient targeting levels for Rituximab.

Relapsed-refractory DLBCL remains a major cause of morbidity and mortality^42^. The patient previously treated with R-CHOP for several cycles developed resistance to further retreatment with Rituximab. According to our preclinical study results, we made a novel regimen of Chidamide with Rituximab for the treatment of relapsed/refractory DLBCL patient with older age and intolerable to chemotherapy. The standard protocol of Chidamide for the treatment of T-cell lymphoma is 30 mg, twice a week. Considering the older age of DLBCL patients and less tolerate to the drug, we reduced dose of Chidamide to 10 mg/day for 2 weeks with Rituximab on the seventh day. In all, 10 mg/day Chidamide equivalents to about 0.5 µM (considering the body weight of patient is 50 kg) which is higher than its IC_50_ of DLBCL cell lines in vitro. The patient was generally tolerable to this treatment regimen, achieved partial response after the first cycle, and responded completely after the third cycle. We found that Chidamide is more effective for the in vivo treatment to human than animal models which require higher doses of Chidamide, according to our experiments and other reports^24,43^.

In summary, this study suggests that Chidamide plus Rituximab might be a potent therapeutic regimen for the treatment of relapsed/refractory DLBCL, although this requires verification in multiple clinical trials. We demonstrate that inhibition of HDACs by Chidamide upregulates expression of CD20 and many other plasma membrane genes. Therefore, it compensates Rituximab-induced loss of CD20. We therefore propose that combination of Chidamide with Rituximab could be an effective regimen for the treatment of relapsed/refractory DLBCL patients who are intolerable to R-CHOP or with poor response to Rituximab.

## Materials and methods

### Study cohorts

Gene expression profiles and clinicla data of 414 DLBCL patients were obtained from the Gene Expression Omnibus (GEO) public databases (GSE10846)^1^ (Supplementary Table 1). Gene expression profiles of 11 DLBCL and 5 normal B cells (including memory B-lymphocyte and naïve pregeminal centre B-lymphocyte) were obtained from GEO public databases (GSE12453)^30^.

### Cell lines and cell culture

DLBCL cell lines Su-DHL6, Su-DHL8 and Su-DHL10 were kindly provided by Professor Anthony Letai (Dana-Farber Cancer Institute). DLBCL cell lines OCI-Ly3 and OCI-Ly7 were kindly provided by Dr. Tian Zhang (Tianjin Medical University Cancer Institute and Hospital). DLBCL cell lines OCI-Ly3, Su-DHL6, Su-DHL8 and Su-DHL10 (refs. ^44,45^) were cultured in RPMI 1640 and OCI-Ly7 in IMDM complete medium at 37 °C in a 5% CO_2_ humidified incubator. All cell lines were recently authenticated by STR profiling and tested for mycoplasma contamination.

### RNA extraction and one-step RT-PCR

RNA was extracted from DLBCL cells using Trizol reagent (Invitrogen) and the RNA pellet was resuspended in 20 μl of DNase-free and RNase-free water. One-step reverse transcriptase-polymerase chain reaction (RT-PCR) from the extracted RNA was conducted according to the manufacturer’s instructions (Takara). RT-PCR was carried out under the following reaction conditions: incubation at 50 °C for 30 min; initial denaturation at 94 °C for 2 min; 30 cycles of amplification at 94 °C for 30 s, 55 °C for 30 s and 72 °C for 1 min/kb. The primer sequences for MS4A1 (CD20) and β-actin were: forward 5′-GCCTGGACTACACCACTCAC-3′ and reverse 5′-AAAACTCCTGAGTCTCCAAGGC-3′ for CD20 and forward 5′-ACACCTTCTACAATGAGCTG-3′ and reverse 5′-CATGATGGAGTTGAAGGTAG-3′ for β-actin, respectively.

### RNA-seq analysis

Three biological replicates RNA samples were collected from OCI-Ly7 and Su-DHL-8 cell lines which were grouped as control, treated with 5 μM Chidamide or 10 μg/ml Rituximab for 24 h. Total RNA was used for RNA-seq analysis. cDNA library construction and sequencing were performed by Beijing Genomics Institute using BGISEQ-500 platform. High-quality reads were aligned to the human reference genome (GRCh38) using Bowtie2. The levels of expression for each of the gene were normalized to fragments per kilobase of exon model per million mapped reads (FPKM) using RNA-seq by Expectation Maximization (RSEM)^46^. Data available at https://www.ncbi.nlm.nih.gov/sra/PRJNA532434, accession number PRJNA532434. Pathway analysis was performed using the GSEA software developed by the Broad Institute^47^. The significance of DEGs was confirmed with the BGI bioinformatics service using the combination of the absolute value of log2-ratio ≥1 and *P* ≤ 0.05 in this research.

### Xenograft mice model of human DLBCL

Two independent cohorts of 6-week-old female BALB/c nude mice were used in this study (no blinding). Ethical approval for this study was obtained from the ethics committee of Tianjin Medical University Cancer Institute and Hospital. Mice were inoculated with 1 × 10^7^ OCI-Ly7 cells in the right flank, followed by daily monitoring. After 3 weeks, the mice which bear tumour (tumour size >100 mm^3^) were randomized (completely randomized by random number table) into four treatment arms (8 mice/group): (1) vehicle control (dimethyl sulfoxide, DMSO, in distilled water, intra-peritoneal, i.p.); (2) Chidamide (3.9 mg/kg i.p.); (3) Rituximab (200 µg/mice i.p.)^48^; and (4) Chidamide (3.9 mg/kg i.p.)+Rituximab (200 µg/mice i.p.). Mice were dosed daily and the treatment efficacy was monitored with daily measurement of tumour size. Mice were sacrificed on the 21st day of the treatment and tumours were collected for further analysis. Treatment responses were evaluated by means of clinically used RECIST criteria; response rates were calculated as the percentage of animals with a complete or partial response^49^.

### Flow cytometry

To determine cell surface expression of CD20 or CD19, intact DLBCL cells were incubated with 2% human anti-globulin antibody for 30 min on ice to block nonspecific bindings and then were stained with anti-CD20-phycoerythrin (PE), anti-CD19-PE antibodies (Supplementary Table 2) or associated isotype controls for 30 min at room temperature in the dark^50^. Cells were then washed with phosphate-buffered saline before analysis by flow cytometry. To determine the percentage of death cells, DLBCL cells was staining with PI (BD Biosciences) and analysed by flow cytometry according to the manufacturer’s protocol.

### Western blotting

Proteins were extracted using CelLytic^TM^ M cell Lysis Reagent (Sigma) supplied with protease and phosphatase inhibitor cocktails (Sigma). Proteins were subjected to 4–12% NuPAGE gels (Thermo Fisher Scientific) and transferred onto PVDF membrane (Sigma) at 20 V for 1 h by a semi-dry transfer. PVDF membrane was blocked with the blocking buffer [5% polyvinyl pyrrolidone PVP, 5% foetal calf serum and 0.1% sodium azide in tris-buffered solution (TBS) containing 0.2% Tween-20] for 30 min and then incubated with primary antibodies (Supplementary Table 2) overnight at 4 °C. Bound antibodies were detected using incubation with horseradish peroxidase-conjugated secondary antibodies in TBST, described previously^50^.

### Cell viability assay

Cell viability was determined by the MTT assay which is based on conversion of MTT into formazan crystals by living cells, and total mitochondrial activity is related to the number of viable cells. Cells were incubated with 0.5 mg/ml MTT for 4 h. Formazan in the cells was then dissolved by adding isopropanol (containing 0.4 N HCl) at a 1:1 dilution and mixed with pipetting. The optical density of soluble formazan was determined at a 570-nm wavelength by a plate reader^51^.

### Immuno-staining and fluorescent microscopy

Cells or tissue on slides were fixed and permeabilized with Cytofix/Cytoperm reagents (BD) and blocked with a buffer containing of 0.1% saponin and 5% donkey serum. Cells were stained with anti-CD20 antibody for 1 h at room temperature. After washing, cells on slides were incubated with Alexa Fluor 546-conjugated secondary at 1:100 dilution in the dark. Slides were then washed for three times, stained with 50 ng/ml DAPI, air-dried at 4 °C in the dark, mounted in ProLong Gold anti-fade reagent (Invitrogen), and viewed under fluorescent microscopy^52^.

### Immunohistochemistry

DLBCL biopsies from xenograft mice model were formalin-fixed, paraffin-embedded and sectioned (4 µm in thickness). The slides were de-paraffinized in xylene and rehydrated through graded ethanol to water before staining. All sections were treated with 5 mM citrate buffer (pH 6.0) for antigen retrieval and with 3% H_2_O_2_ for the inactivation of endogenous peroxidase. After blocking for 30 min, sections were incubated with primary antibodies overnight at 4 °C. After wash, the sections were stained with a secondary antibody for 30 min at room temperature. Diaminobenzidine and hematoxylin were used as a chromogen substrate and for nuclear counterstaining, respectively^53^.

### Treatment of a relapsed/refractory DLBCL patient with Chidamide plus Rituximab

A 75-year-old female was initially diagnosed as primary extra-nodal DLBCL in the small intestine and had surgical resection in May 2010. Following seven cycles of R-CHOP treatment, the patient experienced a grade IV myelosuppression (Supplementary materials for more information). In April 2018, the disease progressed with multiple subcutaneous nodules, failed to respond to Rituximab-associated treatment and diagnosed as relapsed/refractory cutaneous DLBCL. In November 2018, the patient was treated with Chidamide plus Rituximab for three cycles. One cycle lasted for 21 days: Chidamide (Chipscreen Biosciences Ltd, China) 10 mg/day p.o. (days 1–6, 8–14, cycles 1–3) plus Rituximab 375 mg/m^2^ i.v. (day 7, cycles 1–3), rest on the third week (days 15–21) without treatment. The treatment response was clinically evaluated using PET-CT. The written informed consent which was provided by the single patient described for the non-standard therapy (not in a clinical trial) and the usage of patient photos for publication. The ethical approval for this study was made by Tianjin Union Medical Center research ethical committee in accordance with the Declaration of Helsinki.

### Statistical analysis

Data are shown as either mean ± SD or median with interquartile range. The statistical significance was determined by two-tailed Student’s *t*-test, one-way or two-way ANOVA tests with Bonferroni post-tests. The Pearson product–moment correlation method was used to analyse linear correlation between two groups. In all instances, *P* < 0.05 was considered significant, *P < 0.05, **P < 0.01; ***P < 0.001 and ****P < 0.0001. Data analyses were performed with Prism software (version 6.0; GraphPad) and IBM SPSS version 24 for Windows. The categorical analysis divides patients into two cohorts based on of levels of mRNA expression by the X-tile^54^ statistical package. Outcomes, measured from date of diagnosis to event occurrence or date of last follow-up, were the rate of OS^50,55^. Dose–effect curves were calculated with the CompuSyn software (http://www.combosyn.com) and used to generate the combination index (CI, a computational quantitative measure of drug combination effects), reflecting the synergistic activity of the drugs tested CI ≤ 0.1 very strong synergism; CI = 0.1–0.3 strong synergism; CI = 0.3–0.85 synergism; CI = 1.45–3.33 antagonism^56^. PASS (power analysis and sample) software (http://www.ncss.com) were used to estimate the sample size included in animal studies. Assays were set up in triplicate unless otherwise noted in the figure legends.

## Supplementary information

Suppl Tables

Suppl Figure legends

Suppl Figure 1

Suppl Figure 2

Patient information for a case report
