# Supplementary material for: Novel HDAC inhibitor Chidamide synergizes with Rituximab to inhibit diffuse large B-cell lymphoma tumour growth by upregulating CD20
Source: Cell Death Dis. 2020 Jan 6;11(1):20. doi: 10.1038/s41419-019-2210-0 (PMC6944697; doi:10.1038/s41419-019-2210-0)
Supplement: Supplementary file 1 — Suppl Tables [file 41419_2019_2210_MOESM1_ESM.docx]

**Supplementary Tables**

Suppl Table 1. Summary of profiled DLBCL cohorts

| **Study cohort** | **DLBCL1 (Primary test)** | **DLBCL2 (Validation 1)** |
| --- | --- | --- |
| No. of patients | 233 | 181 |
| Specimen type | Frozen | Frozen |
| Study group | Multinational LLMPP | Multinational LLMPP |
| Therapy regimen | R-CHOP | CHOP |
| End point(s) and covariates | OS, IPI, COO | OS, IPI, COO |
| Median age, y (range) | 62 (17-92) | 65 (14-88) |
| **IPI distribution, n(%)** |  |  |
| Low[0,1] | 75 (32) | 62 (34) |
| Low int. [2] | 43 (18) | 45 (25) |
| High int. [3] | 30 (13) | 30 (17) |
| High [4,5] | 24 (10) | 23 (13) |
| **COO, n (%)** |  |  |
| ABC-like | 93 (40) | 74 (41) |
| GCB-like | 107 (46) | 76 (42) |
| Unclassified | 33 (14) | 31 (17) |
| Measurement platform | Affymetrix | Affymetrix |
|  | HG-U133 Plus 2.0 | HG-U133 Plus 2.1 |
| Primary data source | NCBI GSE10846 | NCBI GSE10847 |
| Reference | Lenz et al, 2008 | Lenz et al, 2008 |

Suppl Table 2. List of chemical.

| Name of chemicals | Company | Cat No | Application | Concentration |
| --- | --- | --- | --- | --- |
| Chidamide | Selleck | S8567 | Treatment | 0-40μM; 3.9mg/kg |

Suppl Table 3. List of primary antibodies.

| Name of antibody | Type | Company | Cat No | Application | Dilution |
| --- | --- | --- | --- | --- | --- |
| CD20 | Rabbit | Sigma | HPA014341 | IHC | 1:200 |
|  |  |  |  | IF | 1:100 |
|  |  |  |  | WB | 1:3000 |
| β-actin | Mouse | Sigma | A5316 | WB | 1:10000 |
| GAPDH | Mouse | Santa Cruz | sc-47724 | WB | 1:500 |
| PARP-1 | Mouse | Santa Cruz | sc-8007 | WB | 1:500 |
| HDAC1 | Rabbit | Cell Signaling | 34589 | WB | 1:3000 |
| HDAC3 | Rabbit | Cell Signaling | 57156 | WB | 1:3000 |
| HDAC3 | Rabbit | Cell Signaling | 3949 | WB | 1:3000 |
| Phospho-HDAC3 (Ser424) | Rabbit | Cell Signaling | 85057 | WB | 1:3000 |
|  |  |  |  | IHC | 1:200 |
| HDAC10 | Mouse | Abcam | ab108934 | WB | 1:3000 |
| Acetyl-Histone H3 (Lys27) | Rabbit | Cell Signaling | 8173 | WB | 1:3000 |
| Acetyl-Histone H3 (Lys27) | Rabbit | Abcam | ab177178 | IHC | 1:2000 |
| Histone H3 | Rabbit | Cell Signaling | 4499 | WB | 1:3000 |
|  |  |  |  | IHC | 1:400 |
| PE-CD20 | Mouse | BD | 555623 | FCM | 20μl/test |
| PE-CD19 | Mouse | BD | 555413 | FCM | 20μl/test |
| PE-Mouse IgG1κ isotype Control | Mouse | BD | 555749 | FCM | 20μl/test |
| Rituximab(anti-CD20 chimeric monoclonal antibody) | Human | Roche | MabThera | Treatment | 10μg/ml;  200μg/mice |

IHC indicated Immunocytochemistry; IF indicated Immunofluorescence; WB indicated Western Blotting; FCM indicated Flow Cytometry.

Suppl Table 4. Correlation between expression of MS4A1 and HDACs and cellular localization of HDACs

|  | Correlation with MS4A1 | | HDAC protein Intracelluar location |
| --- | --- | --- | --- |
|  | P value | R |  |
| HDAC1 | <0.0001 | 0.335 | nucleus |
| HDAC2 | <0.0001 | 0.193 | nucleus |
| HDAC3 | 0.027 | -0.108 | nucleus |
| HDAC4 | <0.0001 | -0.302 | cytoplasm/ nucleus |
| HDAC5 | <0.0001 | -0.213 | cytoplasm/ nucleus |
| HDAC6 | <0.0001 | -0.286 | cytoplasm |
| HDAC7 | <0.0001 | -0.224 | cytoplasm/ nucleus |
| HDAC8 | 0.015 | 0.119 | nucleus |
| HDAC9 | 0.0007 | 0.132 | cytoplasm/ nucleus |
| HDAC10 | <0.0001 | -0.243 | nucleus |
