# Supplementary material for: Novel HDAC inhibitor Chidamide synergizes with Rituximab to inhibit diffuse large B-cell lymphoma tumour growth by upregulating CD20
Source: Cell Death Dis. 2020 Jan 6;11(1):20. doi: 10.1038/s41419-019-2210-0 (PMC6944697; doi:10.1038/s41419-019-2210-0)
Supplement: Supplementary file 2 — Suppl Figure legends [file 41419_2019_2210_MOESM2_ESM.docx]

**Suppl Fig. 1 (A)** Kaplan Meier overall survival (OS) curves of patients with DLBCL. Two subgroups, R-CHOP (n=233) treated and CHOP treated (n=181). **(B)** CD20 mRNA expression. DLBCL cell lines, OCI-Ly7, Su-DHL6 and Su-DHL8, were treated with 10µg/ml Rituximab for 24 hours. Levels of CD20 mRNA expression were determined by RT-PCR. β-actin was used as a loading control. **(C)** Determination of CD19 surface expression by flow cytometry. DLBCL cell lines, OCI-Ly7 and Su-DHL8, were treated with Rituximab (10µg/ml) for 24 hours. Cells were stained with anti-CD19-PE antibody or mouse IgG-PE control isotype.

**Suppl Fig. 2 (A-B)** Univarite analysis of HDACs gene expression was performed using Cox regression data analysis in Lenz-R-CHOP and Lenz-CHOP cohort
