# Supplementary figures and images for: Novel HDAC inhibitor Chidamide synergizes with Rituximab to inhibit diffuse large B-cell lymphoma tumour growth by upregulating CD20
Source: Cell Death Dis. 2020 Jan 6;11(1):20. doi: 10.1038/s41419-019-2210-0 (PMC6944697; doi:10.1038/s41419-019-2210-0)

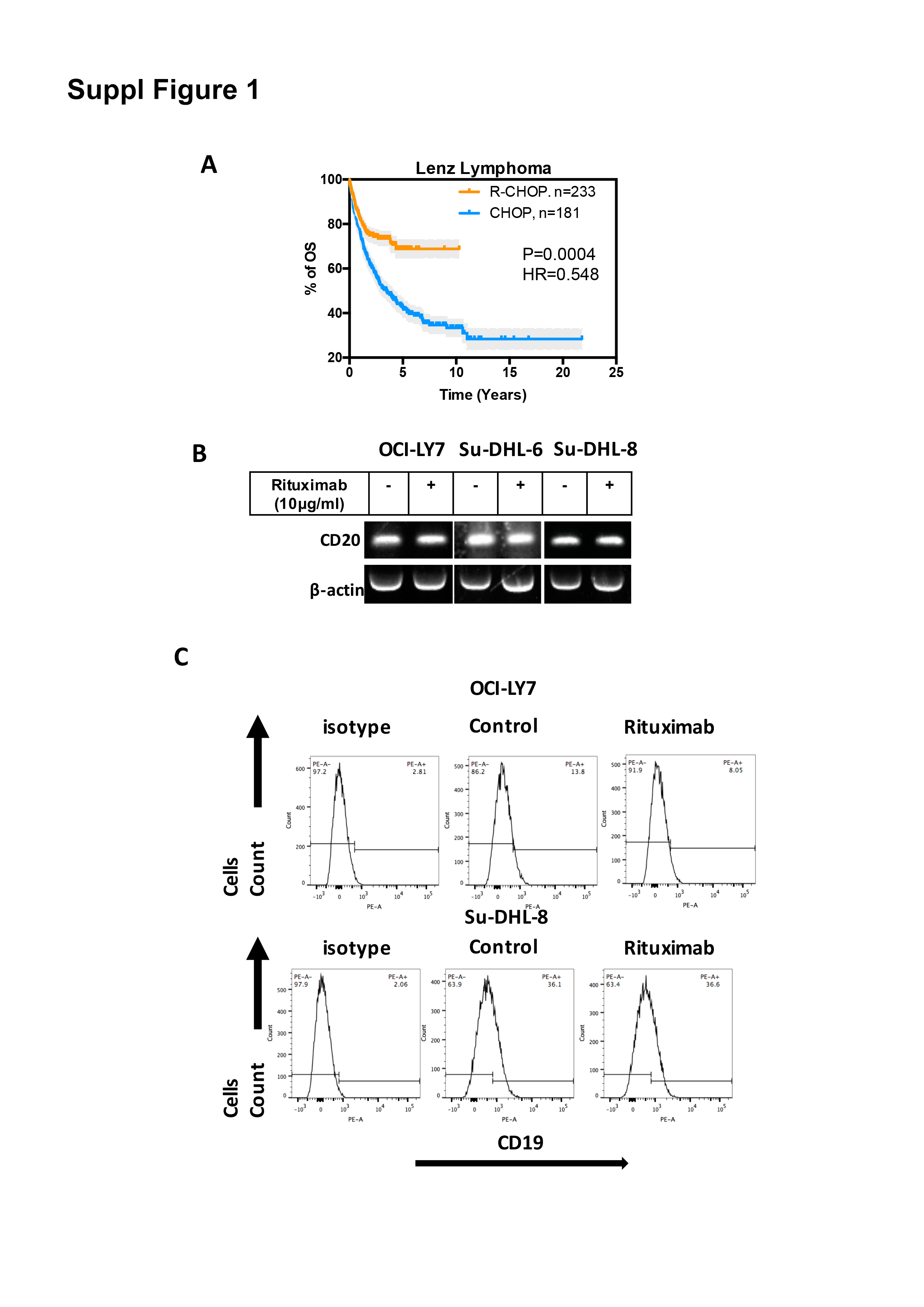

Supplement: Supplementary file 3 — Suppl Figure 1 [file 41419_2019_2210_MOESM3_ESM.tif]

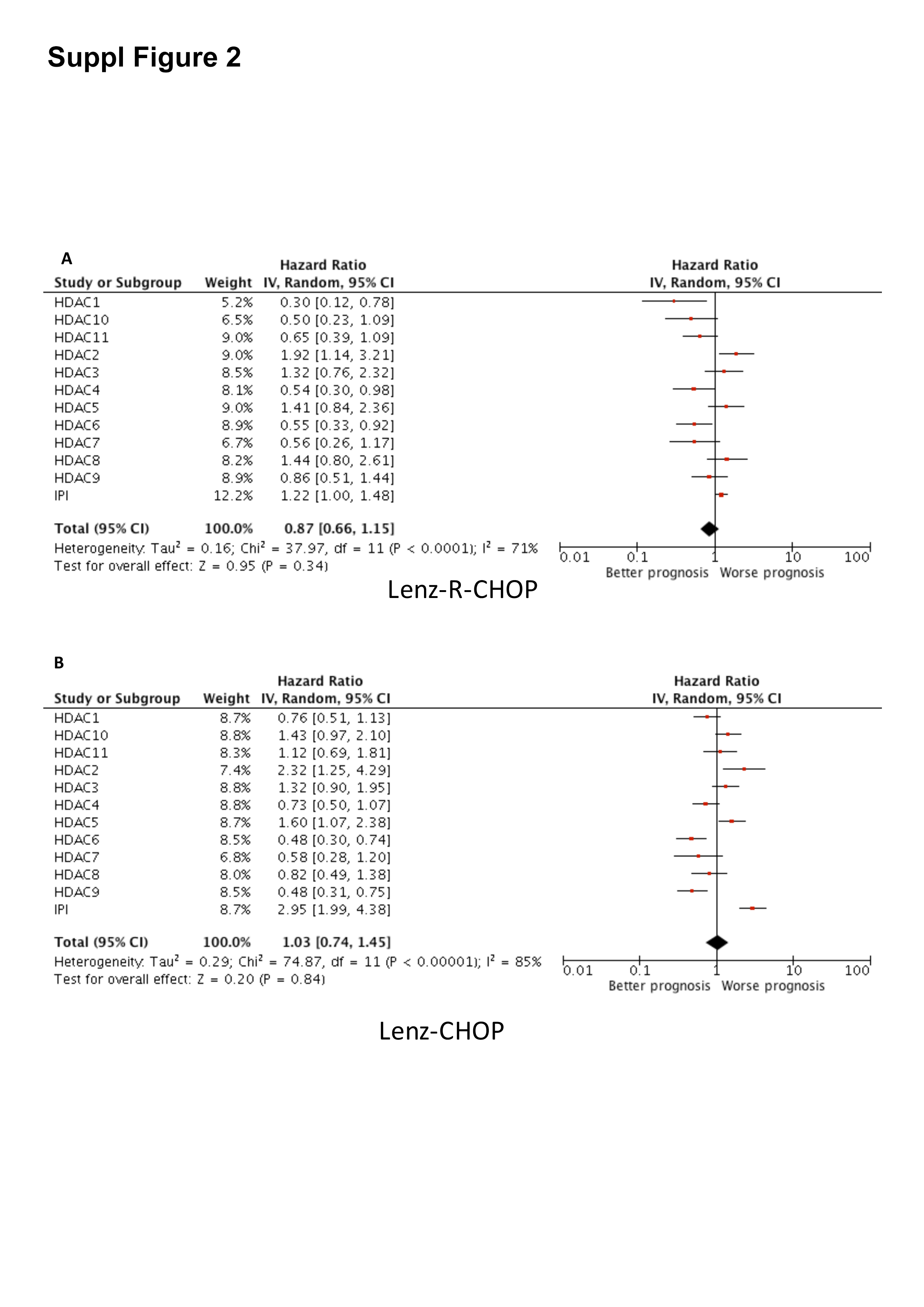

Supplement: Supplementary file 4 — Suppl Figure 2 [file 41419_2019_2210_MOESM4_ESM.tif]
