# Supplementary material for: Novel HDAC inhibitor Chidamide synergizes with Rituximab to inhibit diffuse large B-cell lymphoma tumour growth by upregulating CD20
Source: Cell Death Dis. 2020 Jan 6;11(1):20. doi: 10.1038/s41419-019-2210-0 (PMC6944697; doi:10.1038/s41419-019-2210-0)
Supplement: Supplementary file 5 — Patient information for a case report [file 41419_2019_2210_MOESM5_ESM.docx]

***Patient information for a case report***

A 75 year-old female was initially diagnosed as primary extra-nodal DLBCL in the small intestine and had surgical resection in May 2010. Pathological IHC examination showed that the tumor was CD20+, CD3+, CD10- and Bcl-6-. Following 7 cycles of R-CHOP treatment, the patient experienced a grade IV myelosuppression. In October 2014, she had a nasal tumor, diagnosed as secondary nasal DLBCL after surgical resection with CD20+, CD3+, Ki67+ (80%), Bcl-6-, CD21-, CD50- and she was treated by R-CHOP and R-DOCE for several cycles until a grade IV myelosuppression happened again. Patient was subsequently treated with Rituximab alone for 2 cycles and achieved a complete response. In November 2015, she had multiple nodules in the left breast, with left axillary, double neck, submandibular, and double inguinal lymphadenopathy, treated with R-CHOP for several cycles and achieved as a stable disease. In January 2017, patient underwent a surgical resection to remove the tumor in the left breast, as diagnosed as secondary non-GCB DLBCL with CD20+, CD3+, CD5+, Bcl-2+, Bcl-6-, CD21-, CD10-, cyclin D1-. She was treated with Rituximab plus Gemcitabine and Oxaliplatin, Rituximab plus Bendamustine, Rituximab plus Ibrutinib for several cycles, achieved clinically relevant stable disease. In April 2018, her tumor progressed again with multiple subcutaneous nodules. After treatment with Rituximab plus Bendamustine and Ibrutinib for several cycles the patient failed to respond, therefore it was diagnosed as relapsed/refractory cutaneous DLBCL. In November 2018, the patient was treated with Chidamide and Rituximab for three cycles. One cycle lasted for 21 days. Patient received Chidamide 10 mg/per day po (days 1-6, 8-14, cycles 1-3) plus Rituximab 375 mg/m^2^ iv (day 7, cycles 1-3). The patient had rest on the third week (day 15-21) without treatment. The treatment response was clinically evaluated using PET-CT (Positron Emission Tomography - Computed Tomography). The ethical approval for this study was made by Tianjin Union Medical Center research ethical committee in accordance with the Declaration of Helsinki.
